# Test-retest and intrasession reliability of assisted sprint outcome measurements using a motorized resistance device

**DOI:** 10.3389/fspor.2025.1592707

**Published:** 2025-06-02

**Authors:** Ola Eriksrud, Frederic Westheim

**Affiliations:** Biomechanics Laboratory, Department of Physical Performance, Norwegian School of Sport Sciences, Oslo, Norway

**Keywords:** reliability, motorized resistance technology, assisted sprinting, overspeed, sprint

## Abstract

The aim of this study was to determine reliability of assisted sprint outcome measurements using a motorized resistance device (MRD). A total of 21 participants (16 males and 5 females; age 22.3 ± 3.9 years, body mass 75.2 ± 6.9 kg, height 177.9 ± 6.8 cm) completed two assisted sprint tests on four different test sessions while exposed to an external load (3 kg) provided by the MRD. Outcome variables included time, top speed, maximum acceleration and 5 m split times and speeds. Intrasession and test-retest reliability was assessed using intraclass correlation coefficient (ICC), coefficient of variation (CV), typical error (TE), smallest worthwhile (SWC) and moderate worthwhile change (MDC). For test-retest reliability mostly high to extremely high ICC values (>0.77), and good (<5%) CV values were observed for all outcome measurements. Specifically, all outcome measurements showed high to extremely high relative reliability (ICC ≥ 0.95) except 0–5 m time (0.86) and maximum acceleration (0.77). Good absolute reliability was observed for all outcome measurements (CV range: 0.9–3.7) except maximum acceleration (8.7). Furthermore, most outcome variables were rated as good and ok for SWC. Similar findings were observed for intrasession reliability with very high ICC values for all outcome measurements except maximum acceleration (0.85–0.93) and 0–5 time for session 2–4 (0.88–0.92). Similarly, CV was good for all outcome measurements for all sessions except maximum acceleration (5.7–5.9%). In addition, all outcome measurements had good to ok SWC, except for maximum acceleration. In conclusion, the present study shows that the MRD can obtain reliable assisted sprint outcome measurements and thereby provide coaches and researchers with new opportunities to advance, monitor and evaluate sprint training.

## 1 Introduction

Sprinting is crucial for performance in various individual and team ball sports. Assisted sprinting is one of three common sprint training methods, alongside normal and resisted sprinting, used to enhance speed and acceleration (1). However, resisted sprinting is more widely implemented than assisted sprinting (2), likely due to challenges in individualizing assistance (towing force) and difficulties in quantifying responses and progress. Assisted sprinting can be performed using different equipment, including high-speed treadmills, towing systems [bands, pulleys, motorized resistance devices (MRD)], and environmental conditions such as downhill slopes or tailwinds (2–4). A common misconception is equating assisted sprinting with overspeed or supramaximal sprinting. Specifically, overspeed is exceeding the speed that can be voluntarily achieved in maximal non-assisted sprinting. However, assisted sprinting also includes sprinting at submaximal velocities. In fact, assisted sprinting has been used to target acceleration and top speed (2, 5). Moreover, assisted sprinting may enable more submaximal sprint repetitions by reducing perceived effort (4), potentially due to decreased acceleration demands.

The effects of assisted sprinting have traditionally been classified as acute or chronic, focusing on technique (e.g., temporal characteristics, kinematics), acceleration, and top speed. The observed acute effects on assisted sprinting include decreased time, increased top speed, shorter contact time longer flight time, increased step length, and increased frequency (3, 6–9). Despite these findings, assisted sprinting has not been widely included in technical training programs described in the literature (4), even if it has been suggested that assisted sprinting may be used to produce greater ground reaction forces at comparable speeds (6). The observed chronic effects of assisted sprinting include decreased contact time, increased step frequency, better acceleration (decreased 10 and 20 m times) with a possible effect on top speed (2, 10–12). In addition, pilot data show acceleration improvement in NFL prospects (5). However, no studies have to the authors knowledge specifically examined sprint-specific endurance, despite its perceived importance as a training method (4). Even if the current research is limited (2) it appears that assisted sprinting has a positive effect on performance.

Despite documented performance benefits limited application of assisted sprinting may stem from a lack of suitable equipment to precisely set towing forces or environments (e.g., the absence of a downhill slope). In addition, concerns regarding potential negative effects and injury risks may also contribute to its underutilization. For instance, improper assistance may lead to increased foot touchdown relative to the center of mass, resulting in greater braking forces making it a counterproductive training stimulus (4). Furthermore, overstriding, seeking and finding balance at high speeds, and injury fears are frequently cited as reasons why assisted sprinting is avoided (13).

Recent advancements in MRD technology may facilitate a wider application of assisted sprinting as it allows for individualized prescription and monitoring of assisted sprinting. MRDs have been validated and shown to provide reliable measurements in resisted sprinting (14, 15). However, to the knowledge of the authors no reliability data exists for assisted sprinting. Thus, the purpose of this study was to establish intrasession and test-retest reliability of assisted sprint outcome measurements.

## 2 Methods

### 2.1 Subjects

Sample size calculation (*n* = 19) was based on a minimal acceptable ICC of 0.50, expected ICC of 0.80 based on previous studies using MRD (16), with significance and power level of 0.05 and 0.80 respectively. Twenty-one athletes (16 males: age: 23.0 ± 3.7 years; body mass: 77.3 ± 6.8 kg; height: 179.9 ± 3.7 cm; 5 females: age: 20.0 ± 0.0 years; body mass: 68.6 ± 3.7 kg, height: 171.4 ± 9.5 cm) participated. Participants were recruited from soccer (*n* = 8), handball (*n* = 8), and floorball (*n* = 5), and participated in organized sports since first or second grade (15.3 ± 3.3 years). Inclusion criteria were familiar with ball sports, change of direction movements, linear sprinting and no musculoskeletal injury or illness at time of test sessions limiting maximum effort. The study was approved by the local Ethical committee and the National Data Protection Agency for Research (ref number: 148213) and conducted in accordance with the Declaration of Helsinki. Prior to participation all participants signed a written informed consent after being given a detailed verbal and written explanation of the purpose, procedures and risks associated with participation.

### 2.2 Procedures

Anthropometric measurements (height and body mass) were obtained prior to a standardized warm-up, which included dynamic lower extremity mobility exercises, jogging (forward and backward), butt kicks, front kicks, high knee lifts, side shuffle, carioca, unilateral anterior-posterior and lateral jumps, three progressive sprints (80%, 90–95% of subjective maximal effort) with the last sprint having assistance. Specifically, the subjective effort of these sprints were based on the participants perception of their own top speed. Then, two modified 505 (m505) on each limb both with and without the MRD were performed. In total the warm-up lasted approximately 25 min. The same warm-up was used for all four sessions, and all participants were instructed to standardize their training two days prior to testing. There were 7 days (median, interquartile range: 7 days) between test sessions. All participants were tested at the same time of day (morning or afternoon) based on the first test session using the same footwear.

All test sessions took place in an indoor sports hall at the Norwegian School of Sport Sciences. Tests included assisted and resisted sprinting, resisted anterior and lateral jumps and change of direction tests (modified 505, 10-0-5 and 15-0-5). Only assisted sprint will be presented in this study. Specifically, two assisted sprints were performed with the MRD positioned 65 m away from the starting line. Specifically, the starting line was marked with two cones 1.2 m apart with a finish line at 40 m away from the starting line also marked with cones. The fiber cord from the MRD was attached to the participant via a velcro strap with a handle to a pelvic belt. The participants were instructed to pull up and away on the handle on the velcro strap after having passed the finish line. This to avoid having to decelerate with an additional pulling force. The sprints were initiated from a standing, self-selected split-stance position (left or right foot in front) with the tip of the toe of the front foot placed on the start line. All starts were commenced from a static position, meaning that “leaning backward before rolling forward” was not allowed. After a ready signal was given by the test leader, the participants started on their own initiative. All participants were instructed to complete the 40 m sprint as fast as possible. Recovery time between each sprint was a minimum of 4 min.

### 2.3 Equipment

A portable MRD (1080 Sprint; 1080 Motion, Lidingö, Sweden) was used to provide external resistance and measure time, distance and speed at 333 Hz. The 1080 Sprint has a servo motor (2000 RPM OMRON G5 Series Motor; OMRON Corp., Kyoto, Japan) that is attached to a carbon fiber spool around which a fiber cord is wrapped. The device was positioned on a table at a height of 75 cm to approximately align with participants hip height. Both assisted and resisted load was set to 3 kg since it is a common load used when first exposed to assisted sprinting. From this initial load incremental loading is used to individualize assisted load based on top speed and technical execution. Since the participants were team sport athletes and not previously exposed to assisted sprinting, we kept the assisted load constant at 3 kg for all sessions. Furthermore, the resistance setting was set to isotonic with speed settings set to 2 and 14 m/s for resisted and assisted speeds respectively. The auto start function of the MRD was used (onset of measurement with speed >0.2  m/s) (15).

### 2.4 Data analysis

Assisted sprints were quantified based on time, distance, speed and acceleration. Specifically, top speed, max acceleration with time and average speed for all 5 m splits were analyzed. All outcome measurements are described in Table 1 for clarity. All data were filtered with a fourth order Butterworth filter with a 1.3 Hz cut-off frequency prior to analysis.

**Table 1:** Definitions of all outcome measurements with pros and cons.

Phase	Outcome variables	Definition	Pros	Cons
0–40 m	Time	Time taken to complete the entire 40 m run	Simple overall measurement	Overall measurement with limited information on top speed
	Top speed	The highest speed reached during a specific test	Practical measurement to determine sprint performance (assisted vs. overspeed)	New measurements for coaches based on using split times
	Max accel	0.5 s interval with greatest average acceleration	Information of how the assisted sprint is approached	Might not be relevant if top speed is focus. and how aggressive acceleration is approached is not important
	Best 5 m split time	5 m interval with the lowest time	Automatically find best 5 m split. Split times are commonly used in testing and training	Not comparable to splits based on specific positions
	Best 5 m avg speed	Average speed 5 m interval with lowest time	Top speed based on traditional split	Not comparable to splits based on specific positions
	Best 10 m split time	10 m interval with the lowest time	Automatically find the best 5 m split. Split times are commonly used in sprint testing and training	Not comparable to splits based on specific positions
	Best 10 m split avg speed	Average speed of 10 m interval with lowest time	Top speed based on traditional split	Not comparable to splits based on specific positions
5 m splits	Time	Time to complete 5 m interval	Split times are commonly used in sprint testing and training	Might be obsolete with current technology
	Top speed	Top speed based on filtered data of 5 m interval	Top or maximum speed obtained for a given 5 m interval	Average speed is commonly used with 5 m splits

### 2.5 Statistical analysis

The observed performance of important assisted sprint outcome measurements of session four based on gender (16 males: time: 5.59 ± 0.27 s; top speed: 8.98 ± 0.45 m/s; 0–5 m time: 1.33 ± 0.09 s; 5 females: time: 6.15 ± 0.26 s; top speed: 7.98 ± 0.58 m/s; 0–5 m time: 1.41 ± 0.12 s) was different, but not statistically tested due to group size. Consequently, both genders were grouped for analysis. Statistical analysis was performed using Statistical Package for Social Sciences (SPSS version 24.0, IBM Corp, Armonk, NY, USA) and specifically designed spreadsheets (17). Normality of data was assessed using Shapiro Wilk’s test (*p* < 0.05) and qualitatively by visual inspection of Q-Q plots. Test-retest reliability was calculated based on grand mean of all for test sessions, while intrasession reliability was calculated for each test session. Relative reliability was explored using Intraclass Correlation Coefficient (ICC) (3,1 model) with 95% confidence intervals. The magnitude of the ICC was assessed using the following thresholds: >0.99, extremely high; 0.99–0.90, very high; 0.90–0.75, high; 0.75–0.50, moderate; 0.50–0.20, low; <0.20, very low (18). Absolute reliability was calculated as the typical error of measurement (TE in the unit of the metric) which was then expressed as the coefficient variation (CV, %) with 95% confidence intervals. CV values of >15%, 10–15%, 5–10%, and <5% was used as indication of very poor, poor, acceptable, and good absolute reliability, respectively. In addition, TE was standardized using Cohen’s d effect size principle with magnitudes interpreted as follows: trivial (0.00–0.19), small (0.20–0.60), moderate (0.60–1.20), large (1.20–1.99), very large (2.00–3.99) and extremely large (≥4.00) (18). To assess the usefulness of each outcome measurement TE was compared with thresholds of small (SWC) and moderate (MWC) worthwhile changes or differences (based on 0.2 and 0.6 of between-participant SD). When the TE was smaller than these magnitude thresholds, the test metric was rated as “good” to assess changes/differences of that given magnitude. If the TE was similar to these thresholds, the test metric was rated as “ok.” If the TE exceeded these thresholds, the test metric was rated as “poor,” indicating that only larger changes/differences would be detectable (19).

## 3 Results

Descriptive data for all outcome measurements for the different test sessions with test-retest reliability are presented in Table 2. Absolute reliability (CV) was good for all outcome measurements (0.9–3.7) except Max Accel (8.7). Similar findings were observed for relative reliability (ICC) with very high reliability (0.95–0.99) for all variables except high reliability for 0–5 Time (0.86) and Max Accel (0.77). Similarly, trivial effects were observed for all outcome measurements except small effects for all 5 m top speeds (0.20–0.23), 0–5 m time (0.39) and Max Accel (0.50). A similar pattern was observed with good to ok SWC, except for most 5 m split top speeds, 0–5 m time and Max Accel.

**Table 2:** Descriptive statistics of all test sessions with test-retest reliability of all outcome measurements.

Phase	Outcome variables	Session 1	Session 2	Session 3	Session 4	ICC (95% CI)	CV (95% CI)	TE (95% CI)	Stand TE (95% CI)	SWC	MWC
0–40	Time	5.77 ± 0.49	5.80 ± 0.46	5.77 ± 0.47	5.71 ± 0.36	0.98 (0.97; 0.99)	1.1 (0.9; 1.4)	0.06 (0.05; 0.08)	0.14 (0.11; 0.17)	0.09 (0.07; 0.13)	0.27 (0.21; 0.39)
	Top speed	8.65 ± 0.83	8.56 ± 0.75	8.63 ± 0.79	8.75 ± 0.65	0.98 (0.96; 0.99)	1.4 (1.2; 1.8)	0.12 (0.10; 0.15)	0.16 (0.13; 0.20)	0.15 (0.12; 0.22)	0.46 (0.35; 0.66)
	Max accel	6.34 ± 1.19	6.31 ± 1.04	6.39 ± 1.06	6.36 ± 0.91	0.77 (0.60; 0.89)	8.7 (7.2; 11.5)	0.53 (0.44; 0.67)	0.50 (0.41; 0.63)	0.21 (0.16; 0.31)	0.63 (0.49; 0.92)
	Best 5 m split time	0.58 ± 0.06	0.58 ± 0.06	0.58 ± 0.06	0.57 ± 0.05	0.98 (0.97; 0.99)	1.3 (1.1; 1.7)	0.01 (0.01; 0.01)	0.13 (0.11; 0.17)	0.01 (0.01; 0.02)	0.03 (0.03; 0.05)
	Best 5 m avg speed	8.73 ± 0.84	8.65 ± 0.76	8.71 ± 0.80	8.83 ± 0.66	0.98 (0.96; 0.99)	1.3 (1.1; 1.7)	0.11 (0.09; 0.14)	0.15 (0.12; 0.19)	0.15 (0.12; 0.22)	0.46 (0.35; 0.67)
	Best 10 m split time	1.17 ± 0.12	1.18 ± 0.11	1.17 ± 0.12	1.17 ± 0.09	0.99 (0.97; 0.99)	1.2 (1.0; 1.6)	0.01 (0.01; 0.02)	0.12 (0.10; 0.15)	0.02 (0.02; 0.03)	0.07 (0.05; 0.10)
	Best 10 m split avg speed	8.63 ± 0.81	8.56 ± 0.74	8.61 ± 0.78	8.74 ± 0.66	0.98 (0.97; 0.99)	1.2 (1.0; 1.6)	0.10 (0.08; 0.13)	0.14 (0.11; 0.17)	0.15 (0.12; 0.22)	0.45 (0.35; 0.65)
0–5	Time	1.35 ± 0.13	1.35 ± 0.12	1.35 ± 0.13	1.35 ± 0.11	0.86 (0.75; 0.94)	3.7 (3.1; 4.9)	0.05 (0.04; 0.06)	0.39 (0.32; 0.49)	0.02 (0.02; 0.04)	0.07 (0.06; 0.11)
	Top speed	5.87 ± 0.41	5.81 ± 0.43	5.86 ± 0.37	5.89 ± 0.33	0.96 (0.93; 0.98)	1.6 (1.3; 2.0)	0.08 (0.07; 0.10)	0.20 (0.17; 0.26)	0.08 (0.06; 0.11)	0.23 (0.18; 0.34)
5–10	Time	0.76 ± 0.05	0.76 ± 0.05	0.76 ± 0.05	0.75 ± 0.04	0.99 (0.97; 0.99)	0.9 (0.7; 1.1)	0.01 (0.01; 0.01)	0.12 (0.10; 0.16)	0.01 (0.01; 0.01)	0.03 (0.02; 0.04)
	Top speed	7.19 ± 0.51	7.10 ± 0.58	7.16 ± 0.49	7.23 ± 0.44	0.96 (0.92; 0.98)	1.8 (1.5; 2.3)	0.11 (0.09; 0.14)	0.21 (0.17; 0.27)	0.10 (0.08; 0.15)	0.31 (0.23; 0.44)
10–15	Time	0.67 ± 0.05	0.67 ± 0.05	0.67 ± 0.05	0.66 ± 0.04	0.99 (0.97; 0.99)	0.9 (0.8; 1.2)	0.01 (0.00; 0.01)	0.12 (0.10; 0.15)	0.01 (0.01; 0.01)	0.03 (0.02; 0.04)
	Top speed	7.82 ± 0.60	7.71 ± 0.68	7.81 ± 0.56	7.88 ± 0.51	0.95 (0.91; 0.98)	2.1 (1.7; 2.7)	0.14 (0.11; 0.17)	0.23 (0.19; 0.29)	0.12 (0.09; 0.17)	0.36 (0.27; 0.51)
15–20	Time	0.63 ± 0.05	0.63 ± 0.05	0.63 ± 0.05	0.62 ± 0.04	0.98 (0.96; 0.99)	1.2 (1.0; 1.5)	0.01 (0.01; 0.01)	0.14 (0.12; 0.18)	0.01 (0.01; 0.01)	0.03 (0.02; 0.04)
	Top speed	8.18 ± 0.67	8.08 ± 0.75	8.18 ± 0.65	8.27 ± 0.55	0.95 (0.91; 0.98)	2.2 (1.8; 2.9)	0.15 (0.13; 0.19)	0.23 (0.19; 0.29)	0.13 (0.10; 0.19)	0.40 (0.31; 0.58)
20–25	Time	0.61 ± 0.06	0.61 ± 0.05	0.61 ± 0.05	0.60 ± 0.04	0.98 (0.97; 0.99)	1.2 (1.0; 1.5)	0.01 (0.01; 0.01)	0.13 (0.11; 0.17)	0.01 (0.01; 0.02)	0.03 (0.02; 0.05)
	Top speed	8.41 ± 0.74	8.29 ± 0.81	8.39 ± 0.70	8.50 ± 0.60	0.96 (0.91; 0.98)	2.3 (1.9; 3.0)	0.16 (0.13; 0.20)	0.22 (0.18; 0.28)	0.14 (0.11; 0.21)	0.43 (0.33; 0.62)
25–30	Time	0.60 ± 0.06	0.60 ± 0.05	0.60 ± 0.06	0.58 ± 0.04	0.98 (0.96; 0.99)	1.3 (1.1; 1.8)	0.01 (0.01; 0.01)	0.14 (0.12; 0.18)	0.01 (0.01; 0.02)	0.03 (0.02; 0.05)
	Top speed	8.53 ± 0.77	8.41 ± 0.86	8.50 ± 0.75	8.65 ± 0.62	0.95 (0.91; 0.98)	2.4 (2.0; 3.2)	0.17 (0.14; 0.22)	0.22 (0.19; 0.29)	0.15 (0.09; 0.19)	0.46 (0.35; 0.66)
30–35	Time	0.59 ± 0.06	0.59 ± 0.05	0.59 ± 0.06	0.58 ± 0.05	0.98 (0.96; 0.99)	1.5 (1.3; 2.0)	0.01 (0.01; 0.01)	0.15 (0.13; 0.20)	0.01 (0.01; 0.02)	0.03 (0.03; 0.05)
	Top speed	8.64 ± 0.82	8.47 ± 0.86	8.59 ± 0.78	8.72 ± 0.66	0.96 (0.92; 0.98)	2.4 (1.9; 3.1)	0.17 (0.14; 0.22)	0.22 (0.18; 0.28)	0.16 (0.12; 0.23)	0.47 (0.36; 0.68)
35–40	Time	0.58 ± 0.06	0.58 ± 0.06	0.58 ± 0.06	0.57 ± 0.05	0.98 (0.97; 0.99)	1.4 (1.2; 1.8)	0.01 (0.01; 0.01)	0.13 (0.11; 0.17)	0.01 (0.01; 0.02)	0.03 (0.03; 0.05)
	Top speed	8.61 ± 0.83	8.48 ± 0.87	8.62 ± 0.80	8.74 ± 0.66	0.96 (0.93; 0.98)	2.3 (1.9; 2.9)	0.16 (0.14; 0.21)	0.20 (0.17; 0.26)	0.16 (0.12; 0.23)	0.48 (0.37; 0.69)

Definition and description of all outcome variables are presented in Table 1. Max, maximum; Accel, acceleration; Avg, average; ICC, intra-class correlation coefficient; CI, confidence interval; CV, coefficient of variation; TE, typical error; Stand TE, standardized typical error (light grey, trivial; grey, moderate); SWC, smallest worthwhile change; MWC, moderate worthwhile change [grey shading identifying magnitude (SWC or MWC) of good or ok change/difference].

**Figure 1:**
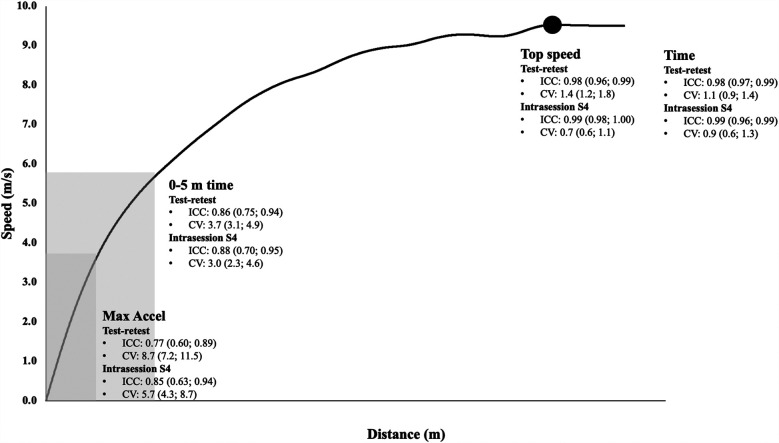
Test-retest and intrasession reliability session four (S4) with ICC and CV (95% CI) of Max accel (darker grey), 0–5 m time (lighter grey), top speed and time.

Intrasession reliability for all test sessions with descriptive data is presented in Table 3. Similar patterns were observed for intrasession as for test-retest reliability. Specifically, absolute reliability (CV) was good for all outcome measurements for all sessions except Max Accel, which ranged from 5.7 to 5.9%. Similar findings were observed for relative reliability (ICC) with very high reliability for all outcome measurements except Max Accel (0.85–0.93), 0–5 Time for session one through four (0.88–0.92). Furthermore, trivial effects were observed for all outcome measurements for all sessions except small effects for Max Accel (0.29–0.46), 0–5 m time (0.20–0.40) and 0–5 m Top Speed session one (0.24). Furthermore, all outcome measurements had good to ok SWC, except for Max Accel and 0–5 m Top Speed session one. A summary of findings for both test-retest and intrasession reliability of session four of key outcome measurements (Time, 0–5 m time, Max Accel and Top speed) are provided in Figure 1.

**Table 3:** Descriptive statistics of all tests with intrasession reliability session 1 through 4.

Phase	Outcome variables	Test 1	Test 2	ICC (95% CI)	CV (95% CI)	TE (95% CI)	Stand TE (95% CI)	SWC	MWC
0–40	Time S1	5.74 ± 0.48	5.75 ± 0.50	0.99 (0.97; 1.00)	1.1 (0.8; 2.4)	0.06 (0.05; 0.09)	0.12 (0.09; 0.18)	0.10 (0.07; 0.15)	0.29 (0.21; 0.46)
	Time S2	5.75 ± 0.47	5.76 ± 0.45	0.99 (0.98; 1.00)	0.8 (0.6; 1.2)	0.05 (0.04; 0.07)	0.11 (0.08; 0.16)	0.09 (0.07; 0.14)	0.27 (0.20; 0.43)
	Time S3	5.76 ± 0.50	5.75 ± 0.48	1.00 (0.97; 1.00)	0.9 (0.7; 1.3)	0.05 (0.04; 0.08)	0.11 (0.08; 0.16)	0.10 (0.07; 0.14)	0.29 (0.22; 0.43)
	Time S4	5.68 ± 0.38	5.70 ± 0.39	0.99 (0.96; 0.99)	0.9 (0.6; 1.3)	0.05 (0.04; 0.08)	0.13 (0.10; 0.20)	0.08 (0.06; 0.12)	0.23 (0.17; 0.35)
	Top speed S1	8.61 ± 0.83	8.64 ± 0.84	0.98 (0.95; 0.99)	1.4 (1.1; 2.1)	0.12 (0.09; 0.17)	0.15 (0.11; 0.21)	0.17 (0.12; 0.26)	0.50 (0.37; 0.77)
	Top speed S2	8.63 ± 0.76	8.61 ± 0.75	1.00 (0.99; 1.00)	0.6 (0.5; 0.9)	0.06 (0.04; 0.08)	0.08 (0.06; 0.11)	0.15 (0.11; 0.24)	0.45 (0.33; 0.71)
	Top speed S3	8.66 ± 0.83	8.64 ± 0.81	0.99 (0.99; 1.00)	0.8 (0.6; 1.2)	0.06 (0.05; 0.09)	0.08 (0.06; 0.11)	0.16 (0.12; 0.24)	0.49 (0.37; 0.72)
	Top speed S4	8.79 ± 0.72	8.78 ± 0.70	0.99 (0.98; 1.00)	0.7 (0.6; 1.1)	0.06 (0.05; 0.10)	0.09 (0.07; 0.14)	0.14 (0.11; 0.21)	0.42 (0.32; 0.64)
	Max accel S1	6.56 ± 1.13	6.29 ± 1.15	0.93 (0.82; 0.97)	5.4 (4.1; 7.8)	0.33 (0.25; 0.48)	0.29 (0.22; 0.42)	0.23 (0.17; 0.36)	0.68 (0.50; 1.08)
	Max accel S2	6.49 ± 1.12	6.30 ± 0.98	0.91 (0.78; 0.97)	5.8 (4.4; 8.5)	0.34 (0.26; 0.49)	0.32 (0.25; 0.47)	0.21 (0.15; 0.33)	0.63 (0.46; 1.00)
	Max accel S3	6.31 ± 0.94	6.45 ± 1.04	0.86 (0.69; 0.94)	5.9 (4.5; 8.8)	0.38 (0.29; 0.56)	0.42 (0.32; 0.61)	0.20 (0.15; 0.29)	0.59 (0.45; 0.87)
	Max accel S4	6.32 ± 0.79	6.34 ± 0.94	0.85 (0.63; 0.94)	5.7 (4.3; 8.7)	0.36 (0.27; 0.54)	0.46 (0.34; 0.68)	0.17 (0.13; 0.26)	0.52 (0.39; 0.78)
	Best 5 m split time S1	0.58 ± 0.06	0.58 ± 0.06	0.98 (0.96; 0.99)	1.5 (1.1; 2.1)	0.01 (0.01; 0.01)	0.14 (0.11; 0.20)	0.01 (0.01; 0.02)	0.04 (0.03; 0.06)
	Best 5 m split time S2	0.58 ± 0.06	0.58 ± 0.06	1.00 (0.99; 1.00)	0.7 (0.5; 1.0)	0.00 (0.00; 0.01)	0.07 (0.05; 0.10)	0.01 (0.01; 0.02)	0.03 (0.02; 0.05)
	Best 5 m split time S3	0.58 ± 0.06	0.58 ± 0.06	0.99 (0.98; 1.00)	0.9 (0.7; 1.3)	0.01 (0.00; 0.01)	0.10 (0.07; 0.14)	0.01 (0.01; 0.02)	0.04 (0.03; 0.05)
	Best 5 m split time S4	0.57 ± 0.05	0.57 ± 0.05	0.99 (0.98; 1.00)	0.7 (0.5; 1.1)	0.00 (0.00; 0.01)	0.08 (0.06; 0.13)	0.01 (0.01; 0.01)	0.03 (0.02; 0.04)
	Best 5 m avg speed S1	8.74 ± 0.84	8.77 ± 0.84	0.98 (0.95; 0.99)	1.5 (1.1; 2.1)	0.13 (0.10; 0.18)	0.15 (0.11; 0.22)	0.17 (0.12; 0.26)	0.50 (0.37; 0.79)
	Best 5 m avg speed S2	8.72 ± 0.78	8.71 ± 0.77	1.00 (0.99; 1.00)	0.7 (0.5; 1.0)	0.06 (0.05; 0.09)	0.08 (0.06; 0.11)	0.15 (0.11; 0.24)	0.46 (0.34; 0.73)
	Best 5 m avg speed S3	8.74 ± 0.85	8.72 ± 0.83	0.99 (0.99; 1.00)	0.8 (0.7; 1.1)	0.07 (0.05; 0.10)	0.08 (0.06; 0.12)	0.17 (0.13; 0.25)	0.50 (0.38; 0.74)
	Best 5 m avg speed S4	8.88 ± 0.72	8.87 ± 0.71	0.99 (0.99; 1.00)	0.7 (0.5; 1.1)	0.06 (0.05; 0.09)	0.09 (0.06; 0.13)	0.14 (0.11; 0.21)	0.43 (0.32; 0.64)
	Best 10 m split time S1	1.17 ± 0.12	1.16 ± 0.12	0.98 (0.96; 0.99)	1.4 (1.1; 2.0)	0.02 (0.01; 0.02)	0.14 (0.10; 0.20)	0.02 (0.02; 0.04)	0.07 (0.05; 0.11)
	Best 10 m split time S2	1.17 ± 0.11	1.17 ± 0.11	1.00 (0.99; 1.00)	0.6 (0.5; 0.9)	0.01 (0.01; 0.01)	0.06 (0.05; 0.09)	0.02 (0.02; 0.04)	0.07 (0.05; 0.11)
	Best 10 m split time S3	1.17 ± 0.13	1.17 ± 0.12	0.99 (0.98; 1.00)	0.8 (0.7; 1.1)	0.01 (0.01; 0.02)	0.10 (0.08; 0.14)	0.02 (0.02; 0.04)	0.07 (0.05; 0.11)
	Best 10 m split time S4	1.14 ± 0.10	1.15 ± 0.10	0.99 (0.99; 1.00)	0.7 (0.5; 1.0)	0.01 (0.01; 0.01)	0.08 (0.06; 0.12)	0.02 (0.01; 0.03)	0.06 (0.04; 0.09)
	Best 10 m split avg speed S1	8.65 ± 0.82	8.68 ± 0.83	0.98 (0.95; 0.99)	1.4 (1.1; 2.0)	0.12 (0.09; 0.17)	0.15 (0.11; 0.21)	0.17 (0.12; 0.26)	0.50 (0.36; 0.78)
	Best 10 m split avg speed S2	8.62 ± 0.76	8.61 ± 0.75	1.00 (0.99; 1.00)	0.6 (0.5; 0.9)	0.05 (0.04; 0.08)	0.07 (0.05; 0.10)	0.15 (0.11; 0.24)	0.45 (0.33; 0.71)
	Best 10 m split avg speed S3	8.65 ± 0.83	8.64 ± 0.81	0.99 (0.98; 1.00)	0.9 (0.7; 1.3)	0.07 (0.05; 0.10)	0.08 (0.06; 0.12)	0.16 (0.12; 0.24)	0.49 (0.37; 0.72)
	Best 10 m split avg speed S4	8.79 ± 0.71	8.78 ± 0.71	0.99 (0.98; 1.00)	0.7 (0.5; 1.0)	0.06 (0.04; 0.09)	0.08 (0.06; 0.13)	0.14 (0.11; 0.21)	0.43 (0.32; 0.64)
0–5	Time S1	1.34 ± 0.13	1.35 ± 0.13	0.97 (0.92; 0.99)	2.0 (1.5; 2.9)	0.03 (0.02; 0.04)	0.20 (0.15; 0.29)	0.03 (0.02; 0.04)	0.08 (0.06; 0.12)
	Time S2	1.34 ± 0.13	1.35 ± 0.11	0.92 (0.80; 0.97)	2.8 (2.1; 4.1)	0.04 (0.03; 0.05)	0.31 (0.24; 0.45)	0.02 (0.02; 0.04)	0.07 (0.05; 0.12)
	Time S3	1.35 ± 0.12	1.34 ± 0.13	0.92 (0.82; 0.97)	2.8 (2.1; 4.1)	0.04 (0.03; 0.05)	0.31 (0.23; 0.45)	0.02 (0.02; 0.04)	0.07 (0.06; 0.11)
0–5	Time S4	1.34 ± 0.10	1.35 ± 0.12	0.88 (0.70; 0.95)	3.0 (2.3; 4.6)	0.04 (0.03; 0.06)	0.40 (0.30; 0.60)	0.02 (0.02; 0.03)	0.06 (0.05; 0.10)
	Top speed S1	5.90 ± 0.40	5.89 ± 0.39	0.95 (0.88; 0.98)	1.6 (1.2; 2.2)	0.09 (0.07; 0.14)	0.24 (0.18; 0.34)	0.08 (0.06; 0.13)	0.24 (0.17; 0.38)
	Top speed S2	5.90 ± 0.37	5.88 ± 0.36	0.99 (0.97; 1.00)	0.8 (0.6; 1.2)	0.05 (0.04; 0.07)	0.13 (0.10; 0.18)	0.07 (0.05; 0.12)	0.22 (0.16; 0.35)
	Top speed S3	5.89 ± 0.38	5.87 ± 0.37	0.98 (0.96; 0.99)	0.9 (0.7; 1.3)	0.05 (0.04; 0.08)	0.14 (0.11; 0.21)	0.08 (0.06; 0.11)	0.23 (0.17; 0.33)
	Top speed S4	5.96 ± 0.36	5.92 ± 0.35	0.98 (0.96; 0.99)	0.8 (0.6; 1.2)	0.05 (0.04; 0.07)	0.14 (0.10; 0.21)	0.07 (0.05; 0.11)	0.21 (0.16; 0.32)
5–10	Time S1	0.75 ± 0.05	0.75 ± 0.05	0.98 (0.94; 0.99)	1.2 (0.9; 1.7)	0.01 (0.01; 0.01)	0.16 (0.12; 0.23)	0.01 (0.01; 0.02)	0.03 (0.02; 0.05)
	Time S2	0.75 ± 0.05	0.75 ± 0.05	0.99 (0.97; 1.00)	0.8 (0.6; 1.1)	0.01 (0.00; 0.01)	0.12 (0.09; 0.17)	0.01 (0.01; 0.02)	0.03 (0.02; 0.05)
	Time S3	0.76 ± 0.05	0.76 ± 0.05	0.99 (0.96; 0.99)	0.9 (0.7; 1.3)	0.01 (0.01; 0.01)	0.13 (0.10; 0.19)	0.01 (0.01; 0.01)	0.03 (0.02; 0.05)
	Time S4	0.74 ± 0.05	0.75 ± 0.05	0.99 (0.98; 1.00)	0.6 (0.4; 0.9)	0.00 (0.00; 0.01)	0.10 (0.07; 0.15)	0.01 (0.01; 0.01)	0.03 (0.02; 0.04)
	Top speed S1	7.20 ± 0.51	7.23 ± 0.51	0.97 (0.93; 0.99)	1.3 (1.0; 1.9)	0.09 (0.07; 0.14)	0.18 (0.14; 0.27)	0.10 (0.07; 0.16)	0.31 (0.22; 0.48)
	Top speed S2	7.21 ± 0.49	7.19 ± 0.48	0.99 (0.98; 1.00)	0.7 (0.5; 1.0)	0.05 (0.04; 0.07)	0.11 (0.08; 0.16)	0.10 (0.07; 0.15)	0.29 (0.21; 0.46)
	Top speed S3	7.19 ± 0.52	7.18 ± 0.51	0.99 (0.98; 1.00)	0.7 (0.5; 1.1)	0.05 (0.04; 0.07)	0.09 (0.07; 0.14)	0.10 (0.08; 0.15)	0.31 (0.24; 0.45)
	Top speed S4	7.29 ± 0.47	7.26 ± 0.46	0.99 (0.99; 1.00)	0.5 (0.4; 0.8)	0.04 (0.03; 0.06)	0.08 (0.06; 0.12)	0.08 (0.06; 0.12)	0.28 (0.21; 0.42)
10–15	Time S1	0.66 ± 0.05	0.66 ± 0.05	0.98 (0.94; 0.99)	1.3 (1.0; 1.9)	0.01 (0.01; 0.01)	0.16 (0.12; 0.24)	0.01 (0.01; 0.02)	0.03 (0.02; 0.05)
	Time S2	0.66 ± 0.05	0.66 ± 0.05	0.99 (0.99; 1.00)	0.6 (0.5; 0.9)	0.00 (0.00; 0.01)	0.08 (0.06; 0.12)	0.01 (0.01; 0.02)	0.03 (0.02; 0.05)
	Time S3	0.66 ± 0.06	0.66 ± 0.05	0.99 (0.99; 1.00)	0.7 (0.6; 1.1)	0.01 (0.00; 0.01)	0.10 (0.07; 0.14)	0.01 (0.01; 0.02)	0.03 (0.02; 0.05)
	Time S4	0.66 ± 0.05	0.66 ± 0.05	0.99 (0.98; 1.00)	0.6 (0.5; 0.9)	0.00 (0.00; 0.01)	0.09 (0.07; 0.13)	0.01 (0.01; 0.01)	0.03 (0.02; 0.04)
	Top speed S1	7.87 ± 0.61	7.88 ± 0.61	0.98 (0.94; 0.99)	1.3 (1.0; 1.9)	0.10 (0.08; 0.15)	0.16 (0.13; 0.24)	0.12 (0.09; 0.19)	0.37 (0.27; 0.58)
	Top speed S2	7.83 ± 0.57	7.83 ± 0.57	1.00 (0.99; 1.00)	0.5 (0.4; 0.7)	0.04 (0.03; 0.06)	0.07 (0.05; 0.10)	0.11 (0.08; 0.18)	0.34 (0.25; 0.54)
	Top speed S3	7.83 ± 0.61	7.84 ± 0.59	0.99 (0.96; 0.99)	1.1 (0.8; 1.6)	0.08 (0.06; 0.11)	0.13 (0.10; 0.19)	0.12 (0.09; 0.18)	0.36 (0.27; 0.53)
	Top speed S4	7.93 ± 0.55	7.91 ± 0.55	0.99 (0.98; 1.00)	0.7 (0.5; 1.0)	0.05 (0.04; 0.08)	0.09 (0.07; 0.14)	0.11 (0.08; 0.16)	0.33 (0.25; 0.49)
15–20	Time S1	0.63 ± 0.05	0.62 ± 0.05	0.98 (0.95; 0.99)	1.3 (1.0; 1.9)	0.01 (0.01; 0.01)	0.16 (0.12; 0.23)	0.01 (0.01; 0.02)	0.03 (0.02; 0.05)
	Time S2	0.63 ± 0.05	0.63 ± 0.05	1.00 (0.99; 1.00)	0.5 (0.4; 0.8)	0.00 (0.00; 0.00)	0.07 (0.05; 0.09)	0.01 (0.01; 0.02)	0.03 (0.02; 0.05)
	Time S3	0.63 ± 0.06	0.62 ± 0.05	0.98 (0.95; 0.99)	1.2 (0.9; 1.7)	0.01 (0.01; 0.01)	0.15 (0.11; 0.22)	0.01 (0.01; 0.02)	0.03 (0.03; 0.05)
	Time S4	0.62 ± 0.05	0.62 ± 0.05	0.99 (0.98; 1.00)	0.7 (0.5; 1.1)	0.00 (0.00; 0.01)	0.10 (0.07; 0.15)	0.01 (0.01; 0.01)	0.03 (0.02; 0.04)
	Top speed S1	8.22 ± 0.68	8.24 ± 0.68	0.98 (0.95; 0.99)	1.3 (1.0; 1.8)	0.10 (0.08; 0.15)	0.15 (0.12; 0.22)	0.14 (0.10; 0.21)	0.41 (0.30; 0.64)
	Top speed S2	8.19 ± 0.64	8.20 ± 0.65	1.00 (0.99; 1.00)	0.6 (0.5; 0.9)	0.05 (0.04; 0.07)	0.07 (0.06; 0.11)	0.13 (0.09; 0.20)	0.39 (0.28; 0.61)
	Top speed S3	8.21 ± 0.70	8.23 ± 0.67	0.99 (0.98; 1.00)	1.0 (0.8; 1.4)	0.07 (0.05; 0.10)	0.10 (0.08; 0.15)	0.14 (0.10; 0.20)	0.41 (0.31; 0.60)
	Top speed S4	8.30 ± 0.60	8.31 ± 0.60	0.99 (0.98; 1.00)	0.7 (0.5; 1.1)	0.06 (0.04; 0.09)	0.10 (0.07; 0.14)	0.12 (0.09; 0.18)	0.36 (0.27; 0.54)
20–25	Time S1	0.60 ± 0.06	0.60 ± 0.06	0.98 (0.96; 0.99)	1.4 (1.0; 2.0)	0.01 (0.01; 0.01)	0.14 (0.11; 0.21)	0.01 (0.01; 0.02)	0.03 (0.02; 0.05)
	Time S2	0.61 ± 0.05	0.61 ± 0.05	0.99 (0.99; 1.00)	0.7 (0.5; 1.0)	0.00 (0.00; 0.01)	0.08 (0.06; 0.11)	0.01 (0.01; 0.02)	0.03 (0.02; 0.05)
	Time S3	0.61 ± 0.06	0.61 ± 0.06	0.99 (0.97; 1.00)	1.0 (0.7; 1.4)	0.01 (0.01; 0.01)	0.12 (0.09; 0.17)	0.01 (0.01; 0.02)	0.04 (0.03; 0.05)
20–25	Time S4	0.60 ± 0.05	0.60 ± 0.05	0.99 (0.98; 1.00)	0.8 (0.6; 1.2)	0.00 (0.00; 0.01)	0.10 (0.08; 0.15)	0.01 (0.01; 0.01)	0.03 (0.02; 0.04)
	Top speed S1	8.44 ± 0.75	8.46 ± 0.75	0.98 (0.95; 0.99)	1.4 (1.1; 2.1)	0.12 (0.09; 0.17)	0.16 (0.12; 0.23)	0.15 (0.11, 0.24)	0.45 (0.33; 0.71)
	Top speed S2	8.41 ± 0.70	8.42 ± 0.69	0.99 (0.98; 1.00)	0.8 (0.6; 1.1)	0.07 (0.05; 0.09)	0.09 (0.07; 0.14)	0.13 (0.09; 0.20)	0.41 (0.30; 0.65)
	Top speed S3	8.42 ± 0.75	8.45 ± 0.73	0.99 (0.98; 1.00)	0.9 (0.7; 1.3)	0.07 (0.05; 0.10)	0.09 (0.07; 0.13)	0.15 (0.11; 0.22)	0.44 (0.34; 0.65)
	Top speed S4	8.55 ± 0.65	8.54 ± 0.65	0.99 (0.98; 1.00)	0.7 (0.5; 1.0)	0.06 (0.04; 0.09)	0.09 (0.07; 0.13)	0.13 (0.10; 0.19)	0.39 (0.29; 0.58)
25–30	Time S1	0.59 ± 0.06	0.59 ± 0.06	0.98 (0.95; 0.99)	1.5 (1.2; 2.2)	0.01 (0.01; 0.01)	0.15 (0.12; 0.22)	0.01 (0.01; 0.02)	0.04 (0.03; 0.06)
	Time S2	0.60 ± 0.05	0.59 ± 0.05	0.99 (0.98; 1.00)	0.8 (0.6; 1.2)	0.00 (0.00; 0.01)	0.09 (0.07; 0.13)	0.01 (0.01; 0.02)	0.03 (0.02; 0.05)
	Time S3	0.60 ± 0.06	0.59 ± 0.06	0.99 (0.97; 1.00)	1.0 (0.8; 1.5)	0.01 (0.01; 0.01)	0.11 (0.09; 0.17)	0.01 (0.01; 0.02)	0.04 (0.03; 0.05)
	Time S4	0.58 ± 0.05	0.58 ± 0.05	0.99 (0.97; 1.00)	1.0 (0.7; 1.5)	0.01 (0.00; 0.01)	0.12 (0.09; 0.19)	0.01 (0.01; 0.01)	0.03 (0.02; 0.04)
	Top speed S1	8.55 ± 0.78	8.59 ± 0.80	0.98 (0.95; 0.99)	1.4 (1.0; 2.0)	0.12 (0.09; 0.17)	0.15 (0.11; 0.21)	0.16 (0.12, 0.25)	0.47 (0.35; 0.75)
	Top speed S2	8.53 ± 0.73	8.53 ± 0.72	0.99 (0.99; 1.00)	0.7 (0.5; 1.0)	0.06 (0.05; 0.09)	0.08 (0.06; 0.12)	0.15 (0.11; 0.23)	0.44 (0.32; 0.69)
	Top speed S3	8.54 ± 0.80	8.56 ± 0.77	0.99 (0.98; 1.00)	0.9 (0.7; 1.3)	0.07 (0.05; 0.10)	0.09 (0.07; 0.13)	0.16 (0.12; 0.23)	0.47 (0.36; 0.69)
	Top speed S4	8.70 ± 0.80	8.67 ± 0.66	0.99 (0.97; 1.00)	0.8 (0.7; 1.1)	0.07 (0.05; 0.11)	0.11 (0.08; 0.16)	0.13 (0.10; 0.20)	0.40 (0.30; 0.60)
30–35	Time S1	0.59 ± 0.06	0.59 ± 0.06	0.98 (0.96; 0.99)	1.5 (1.1; 2.2)	0.01 (0.01; 0.01)	0.14 (0.11; 0.21)	0.01 (0.01; 0.02)	0.04 (0.03; 0.06)
	Time S2	0.59 ± 0.06	0.59 ± 0.05	1.00 (0.99; 1.00)	0.7 (0.6; 1.1)	0.00 (0.00; 0.01)	0.08 (0.06; 0.11)	0.01 (0.01; 0.02)	0.03 (0.02; 0.05)
	Time S3	0.59 ± 0.06	0.59 ± 0.06	0.99 (0.97; 1.00)	1.1 (0.8; 1.6)	0.01 (0.01; 0.01)	0.11 (0.09; 0.17)	0.01 (0.01; 0.02)	0.04 (0.03; 0.05)
	Time S4	0.58 ± 0.05	0.58 ± 0.05	0.99 (0.98; 1.00)	0.8 (0.6; 1.2)	0.00 (0.00; 0.01)	0.09 (0.07; 0.14)	0.01 (0.01; 0.01)	0.03 (0.02; 0.04)
	Top speed S1	8.63 ± 0.82	8.66 ± 0.82	0.98 (0.95; 0.99)	1.5 (1.2; 2.2)	0.13 (0.10; 0.19)	0.16 (0.12; 0.26)	0.16 (0.11, 0.22)	0.49 (0.36; 0.78)
	Top speed S2	8.60 ± 0.75	8.59 ± 0.74	0.99 (0.99; 1.00)	0.7 (0.5; 1.0)	0.06 (0.05; 0.09)	0.08 (0.06; 0.12)	0.15 (0.11; 0.24)	0.45 (0.33; 0.71)
	Top speed S3	8.62 ± 0.82	8.60 ± 0.81	0.99 (0.99; 1.00)	0.9 (0.6; 1.2)	0.07 (0.05; 0.10)	0.08 (0.06; 0.12)	0.16 (0.12; 0.24)	0.49 (0.37; 0.71)
	Top speed S4	8.77 ± 0.71	8.75 ± 0.71	0.99 (0.98; 1.00)	0.7 (0.5; 1.0)	0.06 (0.04; 0.09)	0.08 (0.06; 0.12)	0.14 (0.11; 0.21)	0.43 (0.32; 0.64)
35–40	Time S1	0.57 ± 0.06	0.58 ± 0.07	0.98 (0.96; 0.99)	1.6 (1.2; 2.3)	0.01 (0.01; 0.01)	0.14 (0.11; 0.21)	0.01 (0.01; 0.02)	0.04 (0.03; 0.06)
	Time S2	0.58 ± 0.06	0.58 ± 0.05	1.00 (0.99; 1.00)	0.7 (0.5; 1.0)	0.00 (0.00; 0.01)	0.06 (0.05; 0.09)	0.01 (0.01; 0.02)	0.04 (0.03; 0.06)
	Time S3	0.58 ± 0.07	0.58 ± 0.06	0.98 (0.95; 0.99)	1.4 (1.1; 2.0)	0.01 (0.01; 0.01)	0.15 (0.11; 0.22)	0.01 (0.01; 0.02)	0.04 (0.03; 0.06)
	Time S4	0.57 ± 0.05	0.56 ± 0.05	0.99 (0.97; 0.99)	1.0 (0.7; 1.5)	0.01 (0.00; 0.01)	0.11 (0.08; 0.17)	0.01 (0.01; 0.02)	0.03 (0.02; 0.05)
	Top speed S1	8.64 ± 0.83	8.67 ± 0.85	0.98 (0.95; 0.99)	1.5 (1.1; 2.1)	0.13 (0.10; 0.18)	0.15 (0.11; 0.22)	0.17 (0.12, 0.27)	0.50 (0.37; 0.80)
	Top speed S2	8.62 ± 0.77	8.60 ± 0.76	0.99 (0.99; 1.00)	0.7 (0.5; 1.0)	0.06 (0.05; 0.09)	0.08 (0.06; 0.11)	0.15 (0.11; 0.24)	0.46 (0.33; 0.72)
	Top speed S3	8.64 ± 0.85	8.62 ± 0.82	0.99 (0.98; 1.00)	1.0 (0.8; 1.5)	0.08 (0.06; 0.11)	0.09 (0.07; 0.14)	0.17 (0.13; 0.24)	0.50 (0.38; 0.73)
	Top speed S4	8.77 ± 0.72	8.78 ± 0.70	0.99 (0.98; 1.00)	0.7 (0.5; 1.0)	0.06 (0.04; 0.09)	0.08 (0.06; 0.13)	0.14 (0.11; 0.21)	0.42 (0.32; 0.64)

Definition and description of all outcome variables are presented in Table 1. Max, maximum; Accel, acceleration; Avg, average, ICC, intra-class correlation coefficient; CI, confidence interval; CV, coefficient of variation; TE, typical error; Stand TE, standardized typical error (light grey, trivial; grey, moderate); SWC, smallest worthwhile change; MWC, moderate worthwhile change [grey shading identifying magnitude (SWC or MWC) of good or ok change/difference]. S1, session one; S2, session two; S3, session three; S4, session four.

## 4 Discussion

The present study aimed to assess both intrasession and test-retest reliability of assisted sprint outcome measurements using an MRD. Overall, the observed reliability values were good to acceptable for absolute reliability and high to extremely high for relative reliability. These findings are comparable to or better than those reported for change-of-direction performance (16) and resisted sprint outcome measurements obtained by a MRD (15).

Both resisted and assisted sprint training rely on speed monitoring, which can be managed by adjusting the assisted (pulling) load and controlling the speed at which the athlete is pulled. In this study, an assisted load of 3 kg was applied, with the assisted speed set to 14 m/s, well beyond the top speed capacity of all participants. This methodology was informed by practical coaching experience and common practices in assisted sprinting using MRDs. Speed manipulation is typically achieved indirectly by adjusting the assisted load and monitoring the resulting speed of the athlete. Alternatively, one could prescribe a target assisted speed (e.g., 10 m/s) for the athlete to reach; however, this approach is less commonly used. Coaches have reported that reaching a prescribed speed often results in an inconsistent and less fluid experience, potentially due to inter-step speed fluctuations causing slack in the towing line. Given that the current study maintained a constant assisted load, both absolute and relative intrasession and test-retest reliability were found to be good to excellent (Tables 2, 3). In addition, small worthwhile change (SWC) values were mostly classified as good to ok across all outcome measurements.

The reliability of assisted sprint outcome measurements has direct practical applications, particularly as MRDs are increasingly utilized for assisted and overspeed sprint training across various sports. Monitoring speed is crucial for both training and performance assessment. Based on the present findings, both scientists and coaches can now systematically track speed responses to a given load prescription. This is particularly relevant for evaluating training effectiveness and identifying performance changes over time.

In a recent pilot study, Cecilia-Gallego and co-authors examined the effects of a 10-session overspeed training program (10). Their findings indicated that overspeed training had non-significant effects on top speed, as measured by both unloaded sprints and various overspeed conditions. However, considering that the SWC for top speed in the present study was 0.15 m/s for the test-retest reliability, and that they observed a 0.34 m/s increase in an overspeed condition with a similar assisted load, one could argue that meaningful changes occurred. Furthermore, Clark and co-authors reported an acute increase of 0.9 m/s in average speed during the 30–40 m split when transitioning from free sprinting to assisted sprinting (7 kg assisted load), as measured by dual-beam photocells (20). Although their measurements were obtained using a different method, the 0.9 m/s increase substantially exceeds the MWC for the best 10 m split in the present study (test retest: 0.45 m/s; intrasession: 0.14–0.17 m/s). Additionally, the SWC for top speed across different splits ranged from 0.08 to 0.16 m/s, with good MWC values (range: 0.23–0.48 m/s) for the test-retest condition. Furthermore, good SWC were observed for all 5 m split top speed measurements for intrasession reliability that ranged from 0.07 to 0.17 m/s with one exception, 0–5 m top speed session 1 with MWC of 0.24 m/s. In combination these results suggest that the MRD used in this study can detect small but meaningful changes in performance. This ability allows for precise fine-tuning of assisted load prescriptions and training progressions, as well as the detection of performance adaptations in response to training interventions.

Among the various performance metrics analyzed, early acceleration outcomes—specifically maximum acceleration and 0–5 m time—exhibited the lowest reliability for both intrasession and test-retest reliability. Maximum acceleration showed the lowest ICC (test-retest: 0.77; intrasession: 0.85–0.93) and the highest CV (test-retest: 8.7; intrasession: 5.4–5.9). In contrast, better relative reliability was observed for 0–5 m time (test-retest: 0.86; intrasession: 0.88–0.97) and absolute reliability (test-retest: 3.7; intrasession: 2.0–3.0). This discrepancy may be attributed to how these variables are defined and quantified, as well as the way participants executed the test. Maximum acceleration is defined as the 0.5-s interval with the highest average acceleration (21), which typically occurs at the very start of the sprint. Due to the assisted load, participants may not have consistently exerted maximum effort during the initial steps, instead requiring some time to regulate their effort and speed based on the assisted load. This inconsistency could explain the lower reliability observed for maximum acceleration. However, range of differences for Max Accel for all eight tests was 0.27 m/s^2^, which is slightly greater than the observed SWC values for both test-retest and intrasession reliability (0.17–0.23 m/s^2^), making it useful in both scientific and applied settings. Furthermore, the improved reliability for 0–5 m split time and top speed suggests that participants likely self-regulated their speed by the 5 m mark. In addition, both absolute and relative reliability for 0–5 m time is comparable to that of intrasession reliability for resisted sprint with the same load condition (ICC: 0.81; CV: 2.3) (15). Despite the lower reliability of early acceleration metrics, the observed MWC for 0–5 m time (test-retest: 0.07; intrasession: 0.06–0.08) was smaller than the reported changes in 0–5 yard times (0.12 s) following assisted sprint training in NFL prospects (5), indicating that meaningful improvements may still be detected.

The observed lower reliability of acceleration metrics could also be due to the participants as well as the length of the test (40 m). Specifically, the participants were all from team sports and consequently had not been exposed to assisted sprinting with an MRD. The findings might have been different had track and field athletes been included since they might have been more familiar with the type of stimulus. If acceleration is to be targeted is also raises the question if 40 m is the right distance to be used. It might be that shorter distances and more specific cuing on the acceleration phase would be better for team sport athletes. In the current study the only cue used was to complete the sprint as fast as possible. Since it was 40 m, it could be that team sport athletes were trying different pacing strategies during the acceleration phase. Regardless, reliability of acceleration outcome measurements for both test-retest and intrasession is good, but maybe shorter distances with an emphasis on acceleration should be further explored.

Test-retest reliability data were reported across all four test sessions, which potentially may mask learning effects. Many outcome measurements were included in the current study, but based on how assisted or overspeed sprinting traditionally has been applied one could argue that top speed is an important variable to monitor. This variable is very consistent between sessions with an observed increase of 0.10 m/s from session one to four, which is smaller than TE (0.12 m/s) and SWC (0.15 m/s). Furthermore, time provide an overall impression of performance, which decreased from 5.77 to 5.71 s from session one to four, which is the same as TE (0.06 s), but smaller than SWC (0.09 s). Lastly, the acceleration metrics (Max Accel and 0–5 m time) had minimal and no changes from session one to four, well below TE and SWC (Table 2). Thus, it appears that at the group level measurements are very consistent between sessions.

Regarding performance outcomes, the top speeds recorded in this study were comparable to or lower than those observed in previous research. Given that both male and female participants were included, the present findings (8.61–8.79 m/s) (Tables 2, 3) are consistent with those reported by van den Tillaar, who observed speeds of 7.98 m/s for females and 9.00 m/s for males using a 3 kg assisted load (9). Conversely, other studies have reported higher running speeds. For instance, Clark and co-authors reported a top speed of 10.9 m/s (20) while Gleadhill and co-authors reported 10.14 m/s (6).

### 4.1 Limitations and implications for future research

The present study has identified both limitations and avenues for future research. One key limitation is that only a single assisted condition (3 kg) was assessed. Given that various absolute and relative loading conditions (% body mass) have been used in prior studies (10, 20), evaluating the reliability of different assisted loads would have enhanced the generalizability of the findings. Nevertheless, considering the strong reliability observed for both resisted (15) and assisted sprint measurements in the current study, it appears that MRD-based assessments maintain high reliability across different conditions.

Additionally, while the top speeds recorded in this study among athletes from various ball sports align with findings from some previous studies, they are notably lower than those reported in more elite populations (6, 20). Despite this, the reliability data presented here provide valuable insights for coaches and researchers, particularly in the interpretation of speed measurements. Future studies should explore a broader range of assisted loads and investigate their effects on reliability across different athletic populations to further refine training and performance assessment protocols.

## 5 Conclusion

Assisted sprint outcome measurements derived from data captured by an MRD are reliable. The analysis revealed predominantly high to extremely high ICC values, along with generally good CV values for both test-retest and intrasession reliability across multiple sessions. These findings of assisted sprint measurements offer coaches and researchers valuable opportunities to further evaluate and optimize assisted sprinting testing and training protocols.

## 6 Practical applications

Our findings have practical implications for field-based testing and training in assisted sprinting. Building on the previously established validity and reliability of MRD outcome measurements (14–16) the current results offer valuable insights into interpreting performance data, making real-time adjustments during training sessions, and assessing athletes' responses to training programs.

## Data Availability

The raw data supporting the conclusions of this article will be made available by the authors, without undue reservation.
